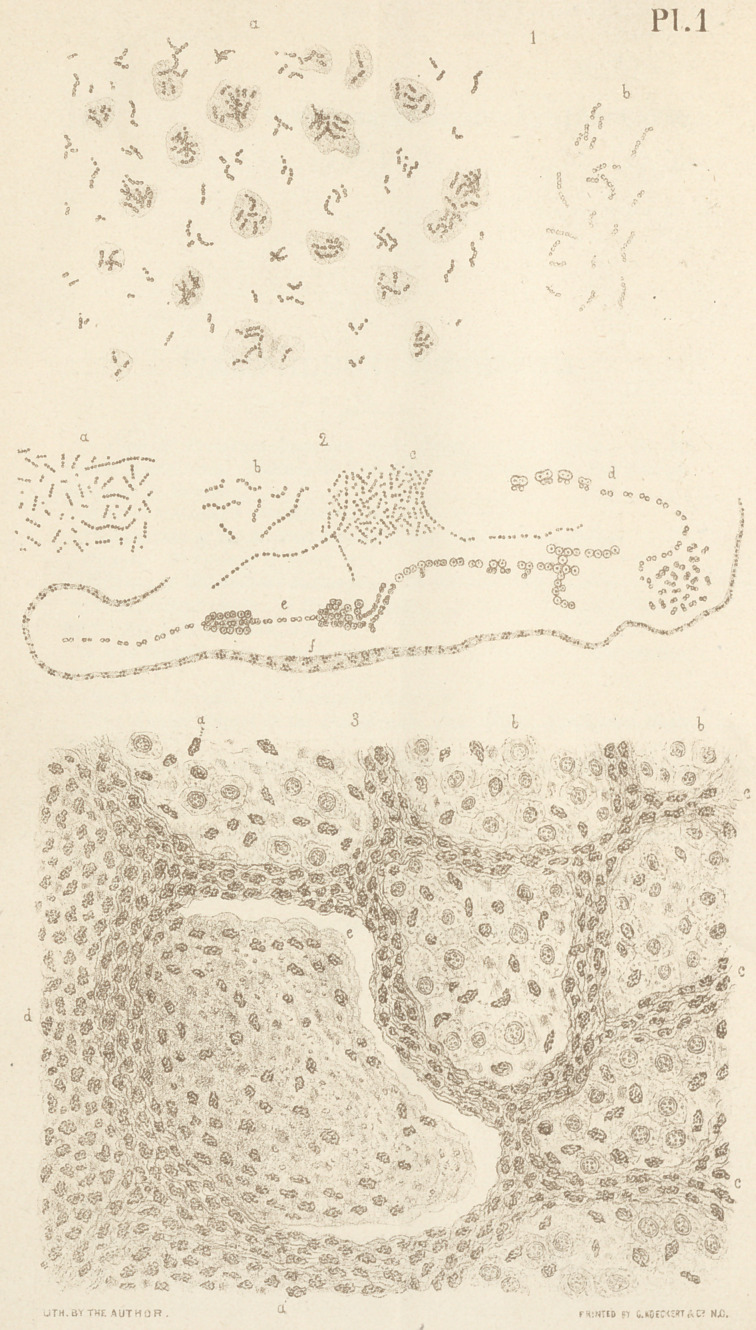# On the Origin and Development of the Bacillus Tuberculosis in the Human Lung, Liver, Spleen, Etc.*The investigations described in this treatise were commenced in the spring of 1883, and continued to the beginning of July, 1884, by which time the author had closely studied the lungs and other organs of twelve or more cases of both miliary and chronic tuberculosis, besides having elicited the principal facts concerning the relation of the bacillus tuberculosis to the tissues of these organs. At this time, however, his expectations of seeing the results of these labors in print during the following winter were dwarfed by a fresh and severe attack of his old enemy, the rheumatism, which kept him confined to his couch for four long and weary months. When, in the ensuing autumn, he was again enabled to resume his accustomed labors, he resolved to render his observations more valuable by the special study of the organs of about fifteen more cases of tuberculosis, and by devoting more time to the preparation of the treatise and the execution of the accompanying illustrations than he had at first intended.

**Published:** 1886-05

**Authors:** H. D. Schmidt

**Affiliations:** Pathologist to the Charity Hospital, New Orleans


THE CHICAGO

### MEDICAL JOURNAL AND EXAMINER.

VOL. LIII. MAY, 1886. No. 5.

### ORIGINAL COMMUNICATIONS.

### ON THE ORIGIN AND DEVELOPMENT OF THE BACILLUS TUBERCULOSIS IN THE HUMAN LUNG, LIVER, SPLEEN, ETC.* *By* H. D. SCHMIDT, M.D., *Pathologist to the Charity Hospital, New Orleans.*

#### INTRODUCTION.

In the following pages I shall state the results of a series of microscopical researches, which, since April, 1883, I have made in regard to the nature of the so-called bacillus tuberculosis of Koch, and to the true relationship of this

* The investigations described in this treatise were commenced in the spring of 1883, and continued to the beginning of July, 1884, by which time the author had closely studied the lungs and other organs of twelve or more cases of both miliary and chronic tuberculosis, besides having elicited the principal facts concerning the relation of the bacillus tuberculosis to the tissues of these organs. At this time, however, his expectations of seeing the results of these labors in print during the following winter were dwarfed by a fresh and severe attack of his old enemy, the rheumatism, which kept him confined to his couch for four long and weary months. When, in the ensuing autumn, he was again enabled to resume his accustomed labors, he resolved to render his observations more valuable by the special study of the organs of about fifteen more cases of tuberculosis, and by devoting more time to the preparation of the treatise and the execution of the accompanying illustrations than he had at first intended.

organism to the tissues of the human tuberculous lung, liver, spleen, etc. These researches may be regarded as a continuation of some others which I made during the summer and autumn of 1882, and the results of which were published in two articles in December, 1882, and February, 1883, in the CHICAGO MFDICAL JOURNAL AND EXAMINER.

Before, however, commencing to speak of these more recent researches on this subject, I deem it proper to present to the reader a few explanatory remarks on the statements concerning the bacillus tuberculosis of Koch, which I made in the two papers just mentioned.

It will be remembered by those who read these papers that, at that time, I failed to demonstrate the bacillus tuberculosis in sections of tuberculous lung, stained by the method which Koch had recommended in his paper, but that by treating these sections with a solution of caustic potash I discovered in the tubercles certain characteristic cells which contained, besides fat-globules and pigment-granules, numerous minute fat crystals. These crystals, representing very minute rods, so closely resembled in form minute bacilli that, as Koch had not mentioned them in his paper, they induced me to regard them as identical with the organisms which Koch had described. This error, as I have already remarked in my second paper, and as I may now repeat, would never have been committed if I had had the opportunity, before taking the above view, of actually seeing Koch's bacillus tuberculosis, or, if Koch, in naming the organism which he had discovered in human tuberculous lungs as well as in the expectorations of tuberculous patients, had considered a little closer the characteristic features of a bacillus, such as they have been defined by Cohn in his classification of the bacteria family. I would not venture to express myself so fully on this subject if I had succeeded to see in only one instance among the thousands of these so-called bacilli tuberculosis, which I have now stained and examined in sections of tuberculous lung and in specimens of tuberculous expectoration, the features displayed which characterize the bacillus form of a bacterium. On the contrary, in every instance I found, not only the form, but all other characteristic features of a so-called sphero-bacterium. Instead of these organisms appearing in the form of minute rods, such as Koch has described them, they represent in reality shorter or longer filaments composed of minute granules. This granular composition of the bacillus tuberculosis of Koch was first observed by Gibbs, and published in August, 1882, in the *London Lancet.* But I must confess that my faith in the true bacillus form of the organism in question, as described by Koch himself, was at that time so great as to incline me to think that the granular filaments, mentioned by Gibbs, did not truly represent the rod-like bacillus tuberculosis of Koch. And accordingly, when I came to observe these granular filaments in tuberculous sputa, treated with a solution of caustic potash, I regarded them as the organisms mentioned by Gibbs, without ever suspecting them to be representations of the veritable bacillus tuberculosis of Koch. Guided then in microscopical examinations by the idea that the bacillus tuberculosis of Koch must show itself in the form of a minute rod, such as its name implies, it must not be wondered that, when discovering in certain degenerating cells of the tubercles minute rod-like fat crystals, which in form closely resembled minute bacilli, the idea arose in my mind that they might be identical with Koch's bacillus tuberculosis. This idea was strengthened not only by the fact that the cells containing these crystalline pseudo-bacilli were met with in the very places where Koch had located his bacillus tuberculosis, that is, along the periphery of the tubercle and outside of the cheesy centre, but moreover by these peculiar cells never having been mentioned in his paper. In the course of this treatise I shall have opportunity to refer again to these cells, which, as far as I know at present, are not identical with the true neoplastic cells of the tubercle, but which, nevertheless, are not without signification in the histological development of this neoplasm. When finally, in January, 1883, the true bacillus tuberculosis of Koch was shown to me in a specimen of tuberculous pustum, such as I mentioned in my second paper on the subject, I found that it was identical with the granular filaments to which Gibbs had referred in his paper, I did not hesitate to acknowledge this fact.

More than three years have passed since my second paper on this subject was published, in which I notified the reader that I should take up the subject again as soon as I should have procured the proper staining material, and not rest until I should have either satisfied myself as to the existence of this pretended parasite, or at least have thrown some light upon the different forms under which it had appeared to different observers. Since that time the bacillus tuberculosis has been a common object of microscopical examination, and thousands of specimens of tuberculous sputum have been stained and examined, and numerous articles written and lectures delivered on the results of these microscopical examinations. But as they were, with a comparatively few exceptions, chiefly made by clinicians for diagnostic purposes, with the view of simply determining the presence or absence of the bacillus tuberculosis in the lungs of their patients, no further light was thrown upon the true origin of the organism, or upon the manner in which it gained access into the human lungs. Nor have the hundreds of thousands of inoculations with tuberculous matter, hitherto made on various kinds of animals, demonstrated anything more than the empirical fact of the presence or absence of the bacillus tuberculosis in the lungs or other organs of the inoculated animal, proving simply the transferability of tuberculosis from one animal to the other. The study of this minute, apparently parasitic organism in the human lungs, the most important locality where it is met with, appears to have been greatly neglected. Those investigators, even, who studied this organism in the tissues of these organs, appeared to have cared mainly to satisfy themselves as to its presence or absence in the latter, for, if their histological labors had been carried on more closely, they would not have failed to observe certain facts which closely bear on the nature and development, and even origin, of these bacteria, and which in the following pages, will form the chief part of my subject.

From the remarks just made it may then be inferred that while much has been done, since Koch first announced his discovery of the bacillus tuberculosis, by way of clinical observation and experimental pathology, to solve the so-called bacillus tuberculosis question, the microscopical research in the field of pathological anatomy has, to the extent of my knowledge, rather been neglected. The reason for this negligence, however, may be easily found in considering that the material for clinical observations, as well as for experimental pathology, is more abundant and more easily obtained than that required for extensive and thorough microscopical researches in pathological anatomy. Every medical practitioner meets with cases of tuberculosis in his daily practice, and as the preparing and staining, as well as the microscopical examination of a little tuberculous sputum, does not require extraordinary skill, nor consume much time, he will have sufficient opportunity of making his own clinical observations on the subject,—while, on the other hand, the material required for extensive investigations in pathological anatomy can only be obtained in connection with the pathological departments of large hospitals. Thus it has happened that so many thousands of specimens of tuberculous sputum have hitherto been examined and discussed in medical societies and journals, while more extensive microscopical studies of thin sections of tuberculous lung, or other organs, have thus far only been made by a comparatively very small number of investigators.

My own labors relating to the bacillus tuberculosis were closely confined to the field of pathological anatomy, for the reason that in this field I am in every respect prepared to do justice to the subject,—not only as regards the abundant fresh pathological material constantly at my disposal, but moreover as regards the proper outfit and assistance for the making of accurate and extensive histological researches. As regards the optical instruments which I use, I may say that during these last twenty-six years I have worked exclusively with first class objectives of the best makers, but especially with those made by the late Mr. Tolles, while the microscopical stands which I have used represent the large patterns made by Mr. Joseph Zentmayer, and constructed for the adaptation of any accessory optical appliance that can facilitate and perfect microscopical examinations. I may state that I have complied with Koch's demand in pursuing my special studies of the bacillus tuberculosis with the assistance of a $\frac{1}{10}$ inch homogeneous immersion objective, which, in the summer of 1884, Mr. R. Tolles made for me for this special work. This objective, constructed not for the purpose of resolving the fine lines of diatoms, but for histological work, with a view of obtaining penetration and perfect definition, is, from what I know, if I may be allowed to say in honor of its maker, the equal to any other objective hitherto made. Besides this objective I also made use of Abbe's illuminating apparatus, which Mr. Zentmayer had adapted to my microscope. Both this apparatus and the homogeneous immersion objective, though they are not absolutely essential, I have reason to say with Koch greatly facilitate an accurate study of the bacillus tuberculosis and its relations. In my own case they proved of great service in confirming the correctness of the observations which I had previously made with dry or water immersion objectives, and a Powel and Lecland's homogeneous achromatic condenser.

In order to properly understand the question of living germs of disease, it is necessary that the reader should be familiar, to a certain extent, with the exact definition of the different forms of bacteria upon which the classification of these organisms is based. Unfortunately the great majority of practicing physicians are unacquainted with the difference of form, or other characteristic features, existing between these minute organisms, which by many physicians are regarded as the original cause of a now considerable number of diseases: for, as they look upon these beings only from a practical point of view, it matters to them very little whether the living germ appears in the form of a sphero-bacterium, or in that of a bacillus. To them the monas tuberculosis of Klebs, the micrococcus tuberculosis of Toussaint and the bacillus tuberculosis of Koch are one and the same in point of signification, as all three have been presented to them as the original cause of tuberculosis. And it is not improbable that the practical physician, in confounding these forms with one another, has come nearer to the truth than the speculating and theorizing bacteriologist. Nevertheless, in treating our subject more philosophically and from a pathological point of view, it is very important that we should be precise in the terms we use and see that they closely correspond with the true definition of the object in question. For this reason, I shall, before proceeding to the description of my observations, make a few passing remarks on the morphological characters of the different kinds of bacteria and their transformation from one form into another.

Although several attempts have been made by divers naturalists to arrange the different kinds of bacteria (Schizomycetes, cleft fungi) according to their form, or other prominent characters, into a number of systematic groups, no perfect classification of these organisms has hitherto been attained. The reason of this failure is to be found in the want of permanency in the various forms under which the bacteria, such as they occur in nature, present themselves to the eye of the observer. The classification which has been most generally used, on account of its convenience for practical purposes, and which for this reason I have formally followed myself, is that of Cohn. It is chiefly based upon the morphological character of the organisms concerned, and consists of the following four groups: 1, *Spherobacteria*, appearing in the form of minute spherical, or oval, cells, the diameter of which is generally less than 0.00 mill. They either appear singly or adhering to one another in pairs, or in chains, formed by a smaller or greater number of cells. When their multiplication by the division of the individual cells becomes very active, they aggregate into masses, or colonies, generally called "zoglœa," which are embedded in a gelatinous material, secreted by the cells. This group embraces but one genus, that of *micrococcus*; 2, *Microbacteria*, embracing, in a narrow sense, the single genus *Bacterium*, represented by short cylindrical, or elliptical cells, which also hang together in pairs. They very rarely appear in the form of chains, though they are met with in the form of zoglœa with abundant intercellular substance; 3, *Desmobacteria*, represented by straight cylindrical cells, usually much longer than wide. They occur isolated, but are usually united in chains. While some of them exhibit active spontaneous movements, others appear motionless. They are represented by the genera *Bacillus* and *Vibrio*; 4, *Spirobacteria*, appearing in the form of cylindrical cells, generally several times as long as wide, and spirally around, similar to a corkscrew. They embrace the genera *Spirillum* and *Spirochæte*. There are a number of other genera of the cleft fungi which, however, may be passed over for the sake of brevity.

When Cohn conceived the above classification of the bacteria, he regarded each of these forms as a distinct seperate genus, the individuals of which would under all circumstances not only reproduce the same forms, but be also endowed with the same physiological properties. In accordance with this view, germ theorists attributed a specific property of producing one or the other specific disease to each form of pathogenic bacteria. More recent investigations, however, have shown that this view is erroneous, and that the forms and other characters of these genera are by no means permanently fixed, but that, on the contrary, one form may, in the course of development, be converted into another. Thus, a bacillus may be developed from a micrococcus, and finally divide again into smaller cells to reassume the micrococcus form. The idea of different forms of bacteria representing but different phases of development of a single natural germ was already advanced, more than ten years ago, by Billroth and some other mycologists; but it was particularly upheld by Nägeli, who pointed out the probability that the various forms, under which the schyzomycetes, or cleft fungi, present themselves, might actually represent but a few natural genera. Nägeli's view that not only the forms of the bacteria, but also their physiological properties, may be altered by artificial cultivation in different nutritive media, was subsequently corroborated by the interesting experiments of Hans Buchner, which experiments, as far as I am able to judge from the perusal of his writings, were made with accuracy, necessary precautions and perseverance. These consisted principally in the conversion of the so-called infectious *bacillus anthracis* into the harmless, non-infectious hay-bacillus, or *bacillus subtilis.* This conversion Buchner effected by supplying the former very freely with oxygen during its cultivation, first in a solution of Liebig's extract of meat, pepton and sugar and afterwards in an infusion of hay. These cultivations were carried through 1,500 generations of the fungus in a space of six months. On the other hand, Buchner converted the non-infectious hay-bacillus, such as it occurs in nature, into the infectious bacillus anthracis by cultivating it, first in egg-albumen and solution of meat extract, and then finishing the cultivation in fresh blood taken from rabbits. He furthermore demonstrated very closely the changes taking place in the form of the bacteria; that is, in the length and width of their individual cells or joints, when cultivated in different nutritive substances. The results which Buchner obtained from his experiments, concerning the convertibility of infectious into non-infectious bacteria, and *vice versa*, and which so conclusively confirmed Nägeli's views regarding the constancy or inconstancy of the forms and physiological properties of these organisms, were, as ought to have been expected, severely criticised by those mycologists who upheld the theory of the specificity of form and property, among whom were Koch and his followers. But the arguments, directed by Koch himself, against the trustworthiness of Buchner's investigations, and calculated to invalidate the facts brought to light by the labors of this investigator, were answered by Buchner in a lengthy but very able treatise, in which, by reviewing and analyzing, in a clear, scientific style, every single point in the charges made against his method of investigation, he deprived the arguments of his opponents of every trace of foundation.

The investigations of Buchner were followed by a series of others, made by Zopf on the highest developed forms of the cleft fungi, viz., Cladothrix, Crenothrix and Beggiatoa, the results of which fully corroborated the view held by Billroth, Nägeli, Buchner, and others. The investigations of Zopf consisted in carefully cultivating the named genera of schizomycetes for the purpose of obtaining each individual germ in a pure condition; that is, isolated from individuals belonging to another genus, or species. The numerous microscopical examinations, made of the fungi concerned during their development, showed him that not only micrococci would grow into bacilli, or the latter be converted into the former, but that even the sword and screw-like forms of the spirilla and spirochæte would appear in one and the same genus during the course of development. The interesting and important results which Zopf obtained from his extensive researches into the development of the cleft fungi and cleft algæ, were published by him in the form of a treatise, fully illustrated by seven beautifully executed lithographic plates * Subsequently to this treatise he published another on the cleft fungi in particular,† in which he treats in a systematic style of the history, anatomy and classification of these organisms. In this little work the subject is treated from the most recent standpoint, based upon the latest observations on the schizomycetes. I do not hesitate to say that I have gained from this little work more definite and positive information, as regards the morphology and nature of bacteria, than from all other works and treatises on the subject I had previously read. Not only this, but certain phenomena which I had observed on different occasions, and for which I was unable to find a satisfactory explanation in other works, were rendered clear to me by the writings of Zopf. These phenomena I observed with bacteria contained in urine, and, as they relate to the subject, I may here briefly state some of them. Bacteria, as is generally known, are very often met with in pathological urine, into which, it is commonly believed, they gain access from the surrounding air, after this fluid has been voided from the bladder. This is probably true in most instances, though, sometimes, I have suspected the reverse, and, in one particular case, in which these organisms were observed in the urine in very great numbers, I convinced myself of their being present in this fluid before its leaving the bladder, by microsopically examining it directly after it was voided. At any rate, from what I know from the numerous

* Zopf. Zur Morphologie der Spaltpflanzen. (Spaltpilze und Spaltalgen.) Verlag von Veit & Comp. Leipzig, 1882.

† Zopf, "Die Spaltpilze."—Nach dem neuesten Standpunkte bearbeitet. Separatabdruck aus der Encyklopædie der Naturwissenschaften. Verlag von Eduard Trewendt, 1883.

specimens of pathological urine which are daily microscopically examined under my supervision at the pathological laboratory of the Charity Hospital, the number of these organisms varies very greatly in the different specimens of urine, collected at one and the same morning, for, while some may be met with in one specimen, and a small number only in the second, they may appear in very great numbers in the third. This fact, which I have observed for years, shows that the absence, or presence in greater or lesser number of the bacteria in the urine, stand in some relationship to the particular condition or constitution of this fluid. The bacterium generally met with is a small micrococcus (fig. 2 *a*) to which the name *micrococcus urinæ* has been applied, though, as it appears to me, it hardly differs in form from those bacteria generally met with in putrefactive liquids. The filaments of the ordinary urine bacteria are straight, and exhibit spontaneous movements. In many specimens of urine, however, a small number of another form of bacteria is met with, the individual filaments of which are seen scattered among the more numerous regular urine bacteria. These (fig. 2 *b*) are distinguished from the latter by their cells being slightly larger and adhering but very loosely to one another, making them appear like rows of beads; they are, moreover, slightly curved, and exhibit no spontaneous movements, resembling, on the whole, the filaments of mycoderma aceti. About two years ago I met these torula-form bacteria in very great numbers in a specimen of albuminous urine, in which the number of the regular urine-bacteria was so small as to be hardly noticed. Desiring to know the ultimate fate of the former I kept this urine for a further study. In re-examining it, accordingly, on the next day, I found these torula-form bacteria considerably increased in number, and, furthermore, that many of them had grown into long filaments (fig.

2 *e*.); on the third day these were also found to have very considerably increased in number. Subsequent microscopical examination revealed another kind of cells (fig. 2 *d*, and *e*.), which appeared to have arisen from the long bacterial filaments, as the bottle containing the urine had remained closed. These last cells appeared to represent true torulæ, multiplying, as the drawing shows, both by budding and by cell-division. A final examination, made some days afterward, showed another formation consisting in a number of long filaments, probably representing gelatinous envelopes, in which a number of small groups of micrococci (fig. 2 *f*.) were observed embedded. It is not improbable that these groups of micrococci originated by the breaking up of larger cells, such as Zopf observed in his investigations.

The above stated observations, which I made on the transformation of bacteria in urine, are too imperfect in themselves to sustain my particular theory, though they are sufficiently correct to corroborate to a certain extent the observations of Zopf and others. My principal object in mentioning them in this place, however, was not to attempt a corroboration of the facts observed by others, but rather to show that valuable information regarding the nature of bacteria may even be gained by casual observations made on these organisms when met with in those liquids in which they naturally occur. A thorough investigation of the whole subject, of course, cannot be made without having recourse to artificial cultivation,—a laborious task, for which I have never had the necessary time and convenience at my disposal.

From what I have said in the foregoing pages, then, we may safely infer that the metamorphosis of one form of bacteria into the other, first pointed out a number of years ago by Billroth and Nägeli, has been proved finally by the labors of Cienkowski, Nealson, Buchner and Zopf to be a fact. The important bearing of this fact on the artificial cultivation of pathogenic bacteria, and with it on the whole question of living disease germs is obvious, as it shows the possibility of changing the form in which these bacteria in reality exist in the human organism, by transferring them to another nutritive substratum. While thus, for instance, the so-called bacillus tuberculosis may exist in the human lungs in the form of a micrococcus, it may be developed into a rod or bacillus form, when artifically cultivated in another nutritive medium.

Having by the foregoing introductory remarks prepared the reader for a better appreciation of the results of my recent investigations concerning the nature of the bacillus tuberculosis, and its relationship to the human lungs and other organs, and for the conclusions which may be drawn therefrom, I shall pass over to the description and discussion of my observations.

### II.

### REMARKS ON THE STAINING OF BACTERIA.

In connection with the demonstration of the bacillus tuberculosis in the thousands of specimens of tuberculosis expectoration, to which I have already referred, a great deal has been said and written on the comparative merits of the different methods for successfully staining this bacterium, and we are justified in presuming that, at the present time, most physicians are familiar with this subject. For this reason I might confine my remarks concerning the staining of thin sections of tuberculous tissue, as well as of tuberculous sputum, to a simple statement of the particular method which I employed, if there were not observed in the different anatomical elements of the tisues certain differences in the degree of staining, which demand a more extended discussion of this subject.

It may be safely asserted that in the special study of pathogenic bacteria the process of staining these organisms plays a most essential part, as it is alone by this process that their presence, as well as their comparative number in the tissues of one or the other organ can be safely determined. When the sections of tissues containing bacteria are very thin and treated with solutions of caustic potassa or soda, the organisms may under favorable circumstances be distinguished by a practiced eye from the tissues in which they are buried, though, at the same time, the chances of failure are sufficiently great to render this mode of preparation unreliable for a thorough study of the subject. Besides, in order to determine safely the presence of bacteria in sections of tissue by the aid of these solutions, the organisms should be present in considerable numbers, as, otherwise, they are very apt to escape observation on account of their resemblance to organic granules, or even to very minute fat globules. I, therefore, cannot but fully endorse the importance which Koch has attached to the staining of his bacillus tuberculosis for determining its presence in tuberculous tissues. The demonstration of certain points concerning the origin and development of these bacteria in the human lungs and other organs, which I observed in the course of my recent investigations, likewise depend upon a perfect staining of the tissues under examination.

When Koch first announced the discovery of his bacillus tuberculosis he claimed as one of the specific properties of this organism the capacity for retaining the aniline color by which it has been stained, in preference to any other color to which it may be subsequently exposed. Thus, if the bacillus has been stained in an alkaline solution of methyl blue—the original solution which Koch used—it would afterward refuse to absorb a concentrated watery solution of vesuvin, whilst all other bacteria, with the exception of the bacillus lepræ, would give up the methyl blue and absorb the brown color of the vesuvin.

This specific property, however, the bacillus tuberculosis only possesses to a limited degree, that is, as long as the animal tissues, in which it is lodged, have not absorbed the second stain; for as soon as these tissues are saturated and stained by the solution of vesuvin, the bacillus likewise will, at the expense of the blue, absorb the brown, though perhaps not as readily as the animal tissues. The failures which I experienced, when in 1882 I first attempted to stain the bacillus tuberculosis by Koch's original method, were due to my ignorance of this fact, for leaving, according to Koch's directions, my sections of tuberculosis lung fifteen minutes in the concentrated solution of vesuvin, I might have expected that every bacillus tuberculosis contained in the sections would be colored brown. But my faith in Koch's directions was so great at that time as to regard the keeping of the sections in the solution of vesuvin for not less than fifteen minutes as the proper test for distinguishing the bacillus tuberculosis from other bacteria. When, in the spring of 1883, I had succeeded in perfectly staining this organism by the superior methods of Ehrlich and Gibbes, and by continued practice gained some experience in the staining of bacteria, I recognized the causes of my former failure. But, being at this time familiar with the appearance of the genuine bacillus tuberculosis of Koch, and remembering that its discovery had been effected by means of the original method of staining of this investigator, I resolved to inquire, at some convenient time, once more into the merits of this method of staining. Accordingly, during the spring of 1884, I put a number of very thin sections of tuberculous lung containing, as I knew from previous stainings with Gibbes' solution of magenta, considerable numbers of bacilli tuberculosis, in Koch's original alkaline solution of methyl blue, and, after having left them there for twenty-four hours, transferred them to a concentrated watery solution of vesuvin. Already, after the lapse of a few minutes, these sections had assumed a dark, dirty, greenish-brown color, which, resisting the action of water or alcohol, had rendered them unfit for examination. As there exist some differences in the aniline preparations, even in those of the same color, and especially in the blue, I came to the conclusion that my methyl blue was not of the same kind as that which Koch had used for his staining, and therefore I made, according to Koch's formula, a fresh solution which I colored with methyl violet. But instead of leaving the sections twenty-four hours in this staining fluid I warmed the latter, such as Koch had also recommended. When perfectly stained the sections were transferred to a concentrated watery solution of vesuvin, in which, however, they were only left three minutes. After their removal from the latter solution they were washed in water, then successively treated with absolute alcohol and oil of cloves, in order to be finally mounted in Canada balsam. The subsequent microscopical examination showed but a very few blue colored bacilli tuberculosis in the sections; the rest had become invisible by having absorbed the vesuvin and thus assumed the same color as the tissue in which they were buried. Therefore, then, I stained another set of sections with the same solution, which, however, had been rendered somewhat stronger by the addition of a little more methyl violet. After having been stained in this solution, the sections were washed in water, and individually treated with the solution of vesuvin. In doing so, I first left each section half a minute in the solution of vesuvin after which it was washed in water and then, for about half a minute, put in alcohol for the purpose of removing that portion of the vesuvin which in all Canada balsam mountings is removed from the sections by the action of absolute alcohol and oil of cloves, with which they are previously treated. From the alcohol the sections were then taken, immersed in water and examined by the aid of a strong loupe, in order to ascertain whether some of the blue staining was still remaining. When this was the case the section was replaced in the solution of vesuvin for one-fourth or one-half minute longer, until, with the exception of a few small bluish spots, it was colored brown throughout. The microscopical examinations of these sections, after mounting in Canada balsam, showed that these bluish spots were produced by numerous bacilli tuberculosis, stained blue by the methyl violet. The same results were obtained when, instead of the sections, tuberculous sputum was stained by this method.

The above described experiments with Koch's original bacteria staining fluid conclusively showed me that the bacillus tuberculosis has no claim for the specific property of successfully resisting the displacement of one aniline color, which it had once absorbed, if subsequently exposed to another color. I can not forbear to think, therefore, that I might, perhaps, have been more successful in my first attempts to stain the bacillus tuberculosis by Koch's original method, in 1882, if I had not so closely followed his own directions; though I do not mean to imply that these directions were entirely wrong, as it is possible that the vesuvin which Koch used for his staining may have differed from that which I obtained, by being less soluble in water.

The original method of Koch for staining bacteria by alkaline liquids has been, to the extent of my knowledge, generally abandoned on account of its being inferior to other methods, particularly to those of Ehrlich and Gibbes. The methyl violet and vesuvin, also, are, for the want of sufficient transparency, inferior to magenta followed by chrysoidin or Bismarck brown, and therefore not suited for accurate research. Nor is the methyl violet sufficiently durable, for when the sections, stained by Koch's method, as above described, were re-examined about two weeks afterwards under the microscope, it was found that the bacilli had completely faded; the methyl violet, by which they had been stained, was observed to be diffused throughout all the surrounding tissue.

The bacillus tuberculosis, as is now generally known, may be successfully stained by other methods and staining liquids, described by various authors.* A number of these I have used myself, but as they all have been commented on by other writers, I shall confine my further remarks on the subject of staining these bacilli to those particular methods which I have finally preferred for my investigations. Of these I may first mention the original method of Ehrlich, in which the aniline oil plays a prominent part. The bacillus tuberculosis, especially when present in tuberculous expectoration, may be successfully stained by Ehrlich's original staining fluid; if there is any disadvantage attending the staining with this fluid, it is its want of permanency, as the bacilli stained by it are liable to fade soon afterward. It is for this reason that I prefer, especially for staining sections of tuberculous tissue, the staining fluid recommended by Gibbes, which in reality is a modification of Ehrlich's, but which, by the alcohol it contains, is capable of holding a greater quantity of aniline oil in solution, while at the same time it is rendered stronger by containing more of the aniline color. This fluid saturates, so to say, the bacteria with color, and thus postpones their fading to a more distant time; for, as far as I am able to judge by experience, the permanency of the staining is proportionate to the quantity of coloring material absorbed by the bacteria. As regards the particular aniline color to be used for the staining, I prefer the magenta, though I have also had very good results with the hydrochloride of rosaniline, or with the methyl violet. While the rosaniline, however, is too light a color for being readily distinguished under the microscope, especially when the organisms are deeply buried in the tissues, the methyl violet has the disadvantage of being not sufficiently transparent and of being too easily dissolved by the absolute alcohol used for dehydrating the sections before mounting in Canada balsam. Thus is may happen that the organisms, though well stained by the methyl violet at first, may appear but faintly or not at all stained after being mounted in Canada balsam. The magenta, therefore, being sufficiently transparent and, from all I know, one of the most stable of the aniline colors generally used, is more suitable for an accurate study of the bacillus tuberculosis and for a permanent mounting of the sections. For the purpose in view, however, it must be of the best quality. The kind which I use is recognized by its solution presenting a purple color, while another inferior kind presents a more scarlet red. The bacilli tuberculosis stained with the former solution present, also, when examined with daylight, a purple color, while those stained with the latter—that is if stained at all—appear more of a scarlet red.

Though I have generally preferred Gibbes' solution for the staining of my sections, I have not always used it in its original strength. In its full strength, such as is expressed by the original formula, this solution is intended for staining the bacillus tuberculosis in a comparatively very short time, according to Gibbes' directions in one-half hour. With this solution the bacteria, whether contained in tuberculous expectoration or in tuberculous tissues, may be very readily stained, for which reason it is very convenient in many cases. In order, however, to stain the bacillus tuberculosis, especially when contained in tuberculous tissues, in a more thorough and permanent manner, I have found it of greater advantage to leave the sections for a longer time in the staining fluid. But as Gibbes' solution, made after the original formula, contains rather too much of the magenta for this purpose, I have, for the staining of my sections, reduced it in strength by taking only one-half of the quantity of magenta put down in the formula. In this modified staining fluid of Gibbes the sections may be left for twenty hours, or even longer. If the weather is very warm, it may happen that during this time the solution is rendered stronger by the evaporation of the alcohol, and that a surplus of the aniline color is found to adhere to the sections, while these, themselves, appear somewhat shrunk in their extent. In cold weather the staining fluid containing the sections may, on the contrary, be slightly warmed on a sand-bath, until a delicate vapor is observed to arise from the surface of the fluid, after which the flame is extinguished, and the sections left to cool off with the sand. When the sections are removed from the staining fluid, they are put in distilled water and left there as long as it will remove any surplus aniline color contained in them; they will then appear soft and pliable and not diminished in size. But if, as I have already remarked, too much of the aniline color—in the present case the magenta—is found to adhere to the sections, while they themselves have shrunk in size, no attempt should be made to put them in this condition in the nitric acid solution for the purpose of discoloring them, as this solution is not capable of removing entirely the surplus aniline, nor of rendering the section again soft and pliable, or of making it expand to its original size. The sections, when in this condition, should be placed in alcohol, which easily removes the surplus of the aniline color without injuring the tissues; as soon, however, as this surplus is removed, perhaps in one-half or one minute, they are transferred from the alcohol into distilled water, in which they will be found to expand to their original size, and from which they may now be transferred to the solution of nitric acid.

In treating the sections with the solution of nitric acid—one part of the acid to two parts of water—as first recommended by Ehrlich, some caution has to be observed, for the reason that, if left too long a time in this solution, the nitric acid will in most cases not only remove the stain of the aniline color from the animal tissues, but also from the bacilli tuberculosis. For my own part, I generally leave the sections in the nitric acid solution *only as long* as they are observed to emit a brown precipitate. As soon as the formation of this precipitate ceases, I quickly transfer the sections from the nitric acid solution to the water, in which they may be noticed to part with some of the magenta previously absorbed and which here presents a purple tint. If the sections are now transferred to clean water and made to rest on a white ground, they still appear colored to a certain extent by the magenta, so that one might be tempted to treat them once more with the nitric acid solution, until they are completely discolored. The error committed in pursuing this course, however, becomes evident by taking into consideration that the absolute alcohol, to the action of which the sections must be necessarily exposed in order to be rendered fit for being mounted in Canada balsam, always dissolves and removes a certain part of the aniline color left in the sections by the nitric acid solution. In most cases, therefore, in which the sections have been completely discolored by the nitric acid, very few or no bacilli will be detected in them after being mounted in Canada balsam.

But as it is desirable to know, before mounting the sections in Canada balsam, whether they may be expected to contain stained bacilli tuberculosis, I expose them at once, and before they are stained with the second color, to the action of alcohol. If after this process the sections still show purplish spots, when examined upon a white ground in water, we may be sure of the presence of stained bacilli and, moreover, that they will no more be discolored by the subsequent action of absolute alcohol, to which they must necessarily be exposed before they can be mounted in Canada balsam. The same course is pursued after the sections have been stained with the second aniline color, be it chrysoidin or Bismarck brown, for, as in the case of magenta, a part of these colors is also lost by the action of the absolute alcohol; while in observing the precaution just mentioned the sections, if not sufficiently stained, may without much inconvenience be replaced in one or the other of these colors, in order to absorb them to the proper degree. For staining the tissues of the sections I prefer Bismarck brown or chrysoidin, for the reason that they are more transparent than vesuvin or methyl blue and therefore better suited for special studies of the bacillus tuberculosis; besides the contrast formed by these colors with the magenta purple of the bacilli is quite sufficient for readily distinguishing these organisms from the tissues containing them. As the length of time for the sections to remain in Bismarck brown or any other color depends, of course, on the strength of the solution it, is difficult to fix exact rules on this point. Each student engaged in the study of tuberculous sections, therefore, should himself determine the time with the aid of his watch, by removing and replacing at certain intervals of time the sections from the staining fluid in order to wash and examine them in water, until the are sufficiently stained; if they remain too long in this fluid the bacilli will, in most instances, also be colored brown.

In the foregoing description of the method which I have generally pursued in staining the bacillus tuberculosis in sections of tuberculous tissues, I have only mentioned the nitric acid solution for decolorizing the animal tissues of these sections. Although this solution answers very well the purpose in view, there is, nevertheless, a considerable disadvantage associated with its action, consisting in the contraction or shrinking of the sections, which renders them less fit for histological studies. In order to avoid this shrinking of the tissues I have had, in many instances, recourse to one of the other agents known to dissolve the aniline color, contained in the fluid, quite as well as the nitric acid. Of these agents I have particularly made use of the formic acid, first recommended, if I remember rightly, by Watson Cheyne. This acid removes the magenta only from the animal tissues, without in the least degree affecting the stained bacilli; the sections, therefore, can be left in it for several minutes without fear of discoloring them too much; the principal advanage in using this acid, however, is that it does not cause the animal tissues to shrink.

With the modification of Gibbes' solution and in the manner described above I have now not only successfully stained many hundreds of sections of the tuberculous tissue and of specimens of tuberculous expectoration, but also various kinds of liquids and infusions containing other bacteria, as, for instance, those met with in urine. The bacteria contained in these liquids were stained as thoroughly as the bacilli tuberculosis. They have retained their color remarkably well, as in some of my preparations, which were stained two years ago, they can still be distinctly seen. The bacilli tuberculosis contained in sections of tuberculous tissue, if thoroughly stained with the magenta solution will also retain the color a long time, almost for a year or even longer.

The bacteria contained in urine or other liquids may be very easily stained in the following manner: a drop of the liquid is put upon a cover-glass and then left to evaporate upon a sand-bath; upon this one or more drops are successively applied and dried in the same manner, until the organic matters contained in the liquid are sufficiently thick for the purpose of staining. But as the stratum thus formed by these matters does not protect the bacteria enclosed within from the action of the nitric acid solution to such an extent as the animal tissues of a section of some tuberculous organ protect the bacilli tuberculosis therein, the cover-glass holding the stratum should be exposed to the action of the nitric acid solution but for a very short time, as otherwise the latter will destroy the aniline color previously absorbed by the bacteria.

Before closing my remarks on the process of staining the bacillus tuberculosis I must point to some important facts which relate to the staining of some of the individual anatomical elements of the tuberculous tissues, and to which I will have occasion to refer when discussing in the following pages my observations on the nature and origin of the bacillus tuberculosis.

At present it appears to be generally known that in the different individual anatomical elements, of which all animal tissues are composed, there exists a considerable difference in the capacity of absorbing coloring matters, such as carmine, hæmatoxylin, etc.; it is thus that the protoplasm of the nuclei absorbs the coloring matter of a staining fluid more readily than that of the cells themselves. This difference in the degree of absorbing coloring materials may also be observed when a tissue passes from a normal into a pathological condition, a phenomenon to which I have already referred several years ago. Thus the protoplasm of an animal cell or that of its nucleus will absorb and retain the coloring material much better when in a state of irritation than when in its normal condition. The same may be said of neoplastic cells absorbing better than the normal cells of old tissues; accordingly in staining a section of a cancerous organ the cancer cells will be found more highly stained than those of the neighbouring healthy tissue. Pathological products, such as fibrinous exudates, the albuminous cylinders met with in the urine of cases of parenchymatous nephritis, etc., also absorb coloring matter in a high degree. In staining with aniline colors vegetable or animal tissues, similar differences are observed in the anatomical elements of these tissues, not only in the degree of power for absorbing the colors, but moreover in that for retaining them. Such an instance we observe in the staining of tuberculous tissues in which the vegetable organisms, the bacteria, retain one aniline color if once thoroughly absorbed better than the animal tissues against a second color subsequently presented to them, while the latter on their part possess a greater power of absorption. Next to the bacteria, it is the nuclei of the tubercle cells which hold the aniline color, once absorbed, against the action of the nitric acid solution, or formic acid, alcohol, etc., as well as against a second color presented to them. This phenomenon manifested by the nuclei of the neoplastic tubercle cells is so constantly observed in sections of tuberculous tissues as to become a reliable test between these nuclei and those of the neighboring normal cells. This difference in the power of retaining the aniline color, however, is not only observed to exist between the nuclei of the tubercle and those of the normal cells, but, moreover, between the nuclei of the individual tubercle cells themselves. In a section of a tubercle, stained with magenta, and discolored with nitric acid solution or formic acid, therefore, a number of nuclei will always be observed to have more or less retained the magenta, while others have lost it. The retention of the staining material by these nuclei indicates the state of irritation which generally precedes that of degeneration. As soon, however, as the nuclei commence to degenerate, their absorbing power diminishes in a degree corresponding to that of the degenerating process, to be lost entirely with the final disintegration of the nuclear protoplasm.

Although the nuclei of the tubercle cells, when in a state of irritation, hold the magenta which they have absorbed against the action of the nitric acid solution, formic acid, etc., they never do it in the same degree as the bacilli tuberculosis. This is readily seen by comparing the particular tint of the magenta which the nuclei present with that of the bacilli tuberculosis; for while the latter, when thoroughly stained and examined microscopically with a daylight illumination, present a distinct purplish color, the former appear not only paler but also of a more reddish tint.

The foregoing remarks concerning the capacity of the nuclei of the tubercle cells to hold, when in a certain condition, the magenta absorbed against the action of nitric or formic acid, I have principally made for directing already in this place the attention of the reader to these facts upon which, as I shall show hereafter, the interpretation of certain phenomena relating to the origin and nature of the bacillus tuberculosis in human organs is based.

### III.

### THE MORPHOLOGICAL CHARACTERS OF THE BACILLUS TUBERCULOSIS.

In order to fully understand my observations regarding the origin and the development of the so-called bacillus tuberculosis met with in human organs and its relationship to the tubercles, the reader should be familiar with the morphological character of this organism, such as they have appeared to me in the course of my microscopical investigations.

In the study of the morphological characters of the bacillus tuberculosis it is important that these organisms should not be obscured by the substance in which they are found embedded. For this reason thoroughly stained specimens of tuberculosis expectoration containing the bacilli are preferable to sections of tuberculosis lung or other organs; though these, if very thin, also answer the purpose. In examining such a specimen of sputum containing numerous bacilli tuberculosis it will be found that they represent shorter or longer filaments which are distinctly composed of minute granules (fig. 1, *a* and *b*). The number of the latter being proportionate to the length of the filament, ranges from two to six, seldom more. While some of these filaments are straight, others, perhaps the greater number, appear slightly curved, presenting in many instances one or two angular bends. In examining, especially on the longer filaments, these curves and bends a little more closely, it will be found that in most instances they occur between two neighboring pairs of granules, suggesting the idea that the whole filament is composed of pairs of granules or diplococci (Billroth). In some instances the original building up of the filaments by diplococci is very striking (fig. 1) and cannot be mistaken. The same angular bends may even be observed on some of the shorter filaments composed only of two diplococci; sometimes even one diplococcus appears to overlap the other (fig. 1, *b*). These angular bends appear to indicate the place where two diplococci have formed a junction by mutual attraction.

Now as regards this apparent building up of the filaments of the bacillus tuberculosis by so-called diplococci, we may point to the fact that all sphero-bacteria or micrococci, such as they are met with in various natural liquids, are artificial infusions when they appear in the form of filaments, are in the same manner built up of pairs of minute cells. Generally these filaments are formed of only two diplococci, comprising two granules; not infrequently, however, the latter by joining one another give rise to shorter or longer chains, of which they themselves represent the individual joints. The building up of these chains by micrococcus filaments and of the latter by diplococci may be easily observed in that form of bacteria generally met with in urine (fig. 2, *a*).

In many specimens of tuberculous expectoration (fig. 1, *a*), but particularly in sections of tubercles undergoing the cheesy metamorphosis or so-called coagulation necrosis (figs. 4, 5, 8 and 9), the bacillus tuberculosis presents itself in the form of groups composed of a larger or smaller number of filaments, which, differing in length, frequently overlie one another in different directions. This peculiar grouping has been mentioned by other authors, but to the extent of my knowledge none of them have ever offered a satisfactory explanation for this phenomenon. As the manner in which these groups of bacilli tuberculosis are formed stands in a certain relation with other facts elicited in my investigations, I shall postpone the remarks which I have to make on this subject to another part of the treatise.

The particular appearance which these minute cells, composing the filaments of the bacilli tuberculosis, present to the eye of the observer, differs, as I have observed, with the degree of intensity of their staining. Thus, if they are highly stained, they will appear to adhere closely to, or be fused with, one another more than is in reality the case. Such an appearance may be caused by a surplus of aniline color deposited in the constrictions existing between the individual cells. For this reason it is quite essential for an accurate study of this mutual relationship of these minute cells or granules, that they should be only moderately stained, as represented in fig. 1, *b*, and, moreover, with a suitable transparent staining material; besides, they should not be too much covered by the purulent matter of the expectoration or by the substance of the tubercles. Examined under such favorable circumstances, then, these granules will hardly ever be found completely fused or exhibiting the straight outlines of a so-called "rod;" even those which form diplococci do not appear completely fused with one another, but in all instances still show their original spherical form, separated by a more or less distinct constriction. The individual diploccoci, forming the filament, however, generally appear not fused into one another (fig. 1, *b*.) A close examination of these minute granules will, furthermore, show that they do not all present one and the same diameter; on the contrary, whilst some of them appear slightly larger than the mean diameter, amounting to about 1-1500 mm., others are smaller. The latter very probably represent young cells or granules, formed by the division of larger ones. It is thus that the smallest cells are always observed at one of the ends of the filaments, where, in many instances, instead of a two-celled diploccocus, three cells will be met with. In some filaments, on the other hand, one of the terminal cells may be seen slightly enlarged and presenting a more oval form. This enlargement and change of form precedes, as I presume, the act of division, and therefore cannot be looked upon as an approach to the bacillus form of a bacterium. In fact, among the many thousands of these filaments which I have closely examined, I have never been able to discover a single one without constrictions, that is, representing the rod-like form of a true bacillus. On the contrary, in every instance, even in those filaments in which the minute cells or granules appear as if fused, I have succeeded in dissolving the minute spherical bodies of which they were originally composed. Examined with my Tolles' homogeneous immersion objective, illuminated with very oblique light, the outlines of these granules are distinctly seen projecting in bas-relief from the filaments.

The facts which I have stated above show, then, that the bacillus tuberculosis, such as it is met with in the human lungs, or other organs, does not present the slightest trace of the bacillus form of a bacterium. A true bacillus, according to Cohn's definition, represents, as I have stated before, a rod without constrictions. Whenever a bacillus divides, the divisions are rectangular, and not preceded by a constriction of the part where the division is to take place. The bacillus subtilis, or hay bacillus, represents a fair type of the bacillus form. This organism is easily obtained from a decoction of hay. In staining a drop of this decoction upon the cover glass with a solution of methyl violet, the bacilli, contained in the former, will readily absorb the aniline color from the liquid, and then show very distinctly the rectangular divisions, which in unstained specimens appear very faintly. It will, furthermore, be found that these marks of division greatly differ from the constrictions which the bacillus tuberculosis represents. Koch has, as far as I know, never mentioned these constrictions and the head-like character or torula-form of his bacillus tuberculosis. On the contrary, he describes the latter as a minute rod (Stæbchen), and, accordingly, regards it as belonging to that group of bacteria formed by the bacilli. The fact of the bacillus tuberculosis being composed of minute granules was first pointed out by Gibbes, several months after Koch's announcement of his discovery. Taking, therefore, into consideration the disadvantages attending Koch's original mode of staining with his alkaline fluid, of which I have spoken before, I am not disinclined to believe that when he first discovered these filaments he was unaware of their torula-form character; if otherwise, I could not comprehend for what special reason he applied the designation "bacillus" to these torula-form organisms, unless he places this designation upon the presence of spores which he stated to have discovered within the filaments. In mentioning these spores in his first paper on the subject, he expresses himself as follows:* "Under certain circumstances, to be mentioned hereafter, the bacilli form already in the animal body spores, of which the individual bacilli contain several, mostly from 2 to 4; they are oval in form and d stributed at equal distances throughout the length of the bacillus."

As far as I can understand these words, they imply that the bacillus, throughout the length of which the spores are distributed at equal distances, represents a rod, and not a row of minute cells. Or did Koch, when placing the organism which he discovered in the tuberculous tissue among the bacilli, perhaps regard the individual minute cells or granules forming the filaments as individual joints of the bacillus? The spore then should be contained in these spherical joints. Or

* Berliner Klinische Wochenschrift, April 10th, 1882, p. 222.

again, are the minute cells themselves regarded as the spores? If so, then I may repeat the question which I have asked once before: Where is the body of the bacillus? For though it is possible that these minute cells, forming the filament, called "bacillus tuberculosis," may, like other micrococcus forms of bacteria, possess a gelatinous envelope, I have never seen them enclosed in a distinct sheath which might represent the wall of the original bacillus. Besides, these cells or granules are mostly spherical, and not oval, as Koch describes his spores; and, moreover, if they represented the spores, then, as there are not special rods to be seen, it would appear as if the bacillus tuberculosis were constantly sporing. Hence, there remains nothing else to presume but that the oval spores mentioned by Koch should be contained in the minute cells of the filament.

Ever since I have become familiar with the veritable bacillus tuberculosis, of Koch, I have sought in vain in various treatises written on this subject for a clear, definite description of the spores, as well as of the bacillus-rod containing them. To the extent of my knowledge there are only a few authors, who have themselves practically investigated the subject under discussion who have mentioned these spores. On account of certain discrepancies existing in their descriptions, as well as for the wants of clearness of expression, however, I have failed to derive the proper satisfaction from the sources. Thus, for instance, Watson Cheyne, who has investigated the nature of the bacillus tuberculosis on an extensive scale, expresses himself as follows*: "The tubercle bacilli vary considerably in length, the longest being about the $\frac{1}{7000}$ inch. They are

* Watson Cheyne "Reports to the Association of the Advancement of Medicine by research on the relation of Micro-organisms to Tuberculosis." Reprinted in New York Medical Abstract, May, 1883, p. 175.

narrow (about 1/5 or 1/6 of their length), more or less rounded at the ends, and they generally present a sort of leaded appearance, clear spots with intermediate stained parts, the rod outside the clear spots being also stained (Plate 1, Fig. 10.) The number of leads in a single rod varies from four to eight, average six. The rods are generally straight, but not uncommonly more or less covered. In tissue they are generally found singly, sometimes in pairs, united at their ends or stuck together side by side. At other times there are two or three lying across each other, the axis of all being more or less in the same direction. In cultivations they are, as a rule, shorter, and stuck together in dense masses. Perhaps their shortness is due to their being broken when spread out on the glass, but I think they are really shorter when growing slowly. (Vide examination of Koch's case of injection into the veins). According to Koch they are motionless." The foregoing quotation from Cheyne's paper represents all that this author at the time says of the morphological characters of the bacillus tuberculosis. While he says nothing of spores, he, nevertheless, mentions the leaded appearance of the organism, and, moreover, "clear spots with intermediate stained parts, the rod outside the clear spots being also stained." In considering the great extent of Cheyne's investigations into the nature of the bacillus tuberculosis, his description of the organism itself is rather meagre. Besides, his expression regarding the clear spots is somewhat obscure, for while he speaks of stained parts intermediate to the clear spots, he finishes the sentence by saying that the rod outside the clear spots is also stained. As far as I am able to understand the last part of the sentence, it is a repetition of the preceding, as otherwise these words might indicate that the stained parts "intermediate to the clear spots" were not identical with the

"rod outside the clear spots." In the above description there is nothing said to the effect that these clear spots represented the spores of the bacillus tuberculosis, though I am inclined to think that the author may have regard them as such, but not being sure of their nature, has hesitated to make any positive assertions. In the same vague manner Formad also speaks of these spores, so that it almost appears as if these authors had been trying to avoid to enter into a more definite description of these bodies.

Two other distinguished authors who also quite extensively investigated the subject under discussion are Messrs. Rabes and Cornil. In order to show certain discrepancies found in the descriptions of the spores of the bacillus tuberculosis, as rendered by different authors, I shall exhibit the view of these investigators by quoting some parts of their description of the morphological characters of the organism in question, as follows:* "They (the bacilli) consist sometimes of small homogeneous rods (batonnets), sometimes of small ovoid or rounded granules placed side by side. They are difficult to see without a coloring re-agent. Nevertheless, in sputa which contain a great number of them, when treated with a weak solution of potassa they are recognized as small hyaline and colorless rods, in which no distinct granules are seen. These small rods then appear larger than in the preparations where they have been colored and dehydrated." A little farther on in the same paragraph the authors continue: "In examining with the No. 10 homogeneous immersion of Verick and the concentrator of Abbé a preparation of phthisical sputum, colored by Ehrlich's method, a larger or smaller number of small rods of various length and form are seen, distinctly alike in diame-

**Rabes* et *Cornil.*—"Note sur les Bacilles de la Tuberculose," and "Journal de l'Anatomie et de la Physiologie normales et pathologi[illegible]e de l'Homme et des Animaux." Juillet, Aout, 1883, p. 456.

ter, some of them homogeneous, colored aniline red or slightly violet, the others formed throughout their whole length by small colored granules. In the sputa of one of our patients, the lungs of which were hollowed out by large cavities, a considerable quantity of bacilli was met with, about one hundred in the field of the microscope with an amplification of 500 diameters. The greater part of these small rods contained small granules placed side by side. We have left this sputa in a tube closed by a cork for three weeks. These sputa, under the influence of putrefaction, had lost their mucus consistence. The colored preparations have then shown to us that almost all the bacilli were solely composed of small colored granules; and they have appeared to us more numerous than in the sputa examined immediately after expectoration. We have drawn (see fig. 12, plate XXIV.) these bacteria left to themselves in the sputa during ten days. It can be seen that the greater part among them are composed of slightly elongated or spherical granules, arranged side by side. In examining closely one of these bacilli with the aid of the homogeneous immersion objective No. 10, and with a high ocular, the borders of the small rods, which are rectilinear and parallel, may be distinctly determined, and it is seen that the colored granules, which probably are spores, are seated in the interior of the small rod."*

---

*I cannot forbear to remark that it is impossible to form a correct idea of the morphological characters of the bacillus tuberculosis from those bacilli represented in figure 12, plate XXIII, to which the authors refer in their text. For although this figure is marked in their "Explanation" of the plates as magnified 1000 diameters, the bacilli are represented therein not larger than those in figure 10 on the same plate, marked as magnified only 150 diameters. The granules of the true bacillus tuberculosis when magnified 1000 diameters, present a diameter at least three times as large as represented in the above mentioned figure. The latter, therefore, has missed its purpose of illustrating the morphological characters of the bacillus tuberculosis.

Two years afterwards, in a lecture delivered by Cornil on Pulmonary Tuberculosis,* this investigator makes the following remarks: "The typical bacilli of tuberculosis, as observed in sputum and lung sections colored by Ehrlich's method, appear entirely homogeneous, or formed of small beads adhearing end to end."

"These small beads are considered the spores of bacilli more through analogy with other bacteria than by a true demonstration. We do not know, however, whether this parasite found in tubercular products be not the offspring of parents having an entirely different morphological appearance," etc.

These words are clear enough to show that at present this author also entertains some doubts as to the existence of the so-called spores of the bacillus tuberculosis, he admits even that they never had been truly demonstrated.

The best information concerning the spores of the bacillus tuberculosis which I have thus far obtained, was from a review written by Litten on the "Ætiology of Tuberculosis," by Koch, published in the "Mittheilungen aus dem Kaiserl. Gesundheitsamt." In order to render my arguments on this subject clearer to the reader, I shall quote from this condensed account on the ætiology of tuberculosis the part which treats of these spores, found on page 262 of the "Deutsche Medizinalzeitung," March 24th, 1884. It reads as follows: "A further *very important property of the tubercle bacilli is the formation of spores.*" It was Koch who first observed the appearance of shining bodies in hay-

---

**New Orleans Medical and Surgical Journal.*—A lecture by Dr. Cornil, Prof. of Pathological Anatomy, etc., Paris. Reported by Dr. E. Laplace, of New Orleans, Sept. 1885.

bacilli, which remain after the disintegration of the bacilli and are capable of germinating anew into bacilli. For the latter reason Cohn regarded them as the fruit-form of the bacilli, and designated them spores. Tinged with aniline colors these bacilli appear in the microscopic picture with short articulations, mostly consisting of two articles of which the single ones are darkly colored throughout, still resembling bacilli free from spores. In many articles, however, the appearance of a light spot is observed, which gradually increases in size while the colored contents of the article are more and more retracted to both ends, and while the sides are only still bounded by fine lines representing the outlines of the article. The light space in the interior of the bacillus article is *the spore*, which is not distinguished by its lustre but by its remaining free from coloring matters. The articulation, of course, does not always appear so sharply defined in opposition to one another as in the bacilli pictured by Koch upon the photograms (No. 76 of volume I). In many varieties of bacilli, as, for instance, in those of splenic fever, the articles appear closely opposed to one another and form a continuous thread which contains the uncolored spores at equal distances. The formation of spores in the tubercle bacilli takes place in the same way. The mutual connections of the latter remain preserved and they do not break up into single articles, but in each article a light body is formed so that the bacillus, after being stained, resembles a dark minute thread, interrupted by light egg-shaped places. With the use of the strongest systems and a considerable amplification, it may be ascertained that the spore containing bacillus presents, only on a smaller scale, the same picture as the spore-containing bacillus of splenic fever. The spores are egg-shaped and bordered on their margins by a fine colored line, while their number amounts from 2 to 6 in a single bacillus. As every single spore occupies an article the number of the articles of the bacillus, that is the simple elements of which the latter is constructed, may be inferred from the number of spores. When a substance with spore-containing tubercle-bacilli is examined in a feebly refractive liquid medium the bacilli appear then provided with highly lustrous bodies; accordingly the latter cannot be vacuoles or simple empty spaces contained in the protoplasm of the bacillus, but must represent genuine spores. Upon the accompanying figures of the spore-containing tubercle-bacilli the latter, in order to represent the spores, had to be drawn larger than they in reality appeared by the respective amplification used."

If I understand the meaning of the preceding quotation correctly, Koch describes his bacillus tuberculosis as presenting the form of a true rod, in which, as the writer of the review expresses himself, "the mutual connection of the articles of which the bacillus is composed remains preserved." The rods do not "break up into single articles, but in each article a clear body is formed, so that the bacillus, when stained, resembles a minute dark thread, interrupted by light egg-shaped spaces." He futhermore states that the bacillus tuberculosis, when containing spores, presents the same picture, only on a smaller scale than the spore-containing bacillus anthracis. Now, as far as I know, and as all drawings of the latter—Koch's own "Untersuchungen ueber die Ætiologie der Wundinfections Krankheiten" (fig. 4, 13 and 14) included—show, the articles of this bacillus are characterized by square ends and do not represent spherical bodies. In the same manner has Koch represented in the form of a rod the bacilli he discovered in the blood of septicæmic mice as well as those he met with in the erysipelatous ear of a rabbit (l. c. figs. 1, 2, 3 and 12). This difference of form shows plainly that the bacillus tuberculosis *does not* present on a smaller scale the same picture as the bacillus anthracis.

During the whole course of my investigations into the nature of the bacillus tuberculosis I have closely examined the beaded filaments representing it, with the view of discovering the oval spores in the individual cells of the latter, —presuming, of course, that these cells represented the individual cells of the bacillus,—but my efforts made in this direction have been in vain. My examinations, as I have already stated before, were made under the most favorable circumstances,—that is, with Tolles' 1–10 homogeneous immersion objective, Abbé's illuminator, and the finest daylight that can be obtained from a clear southern sky. To these advantages, however, some one might perhaps raise the objection of my objective not being sufficiently high in magnifying power for discovering the clear oval spaces representing the spores in these minute cells. My reply to such an objection would be that the correction of this objective is so perfect as to render it capable of bearing not only the eyepiece B, but even C, without any more loss of definition than that caused by the loss of penetration attending all high eye-piecing. The amplification, however, obtained with my eye-piece B, as may be judged from my figures of the bacillus tuberculosis accompanying this paper, is more than sufficient to show any clear unstained oval spot or space in the cells of the stained filaments, if really contained therein. A still higher amplification, without much loss of definition, I might obtain by making use of my Tolles' amplifier. But no advantage is gained in making these examinations with very high powers, for the reason that too much penetrative power is lost by the shortness of the focus of the latter to clearly distinguish the unstained spores in the interior of the minute cells of the bacillus.

Not having been able, then, to discover anything in the form of a spore in the granular filaments of the bacilli tuberculosis, met with in the tuberculous organs and sputa of man, and not feeling inclined to disregard the statements of Koch, I can only presume that the spores which this investigator describes he must have observed in those filaments of bacilli tuberculosis which he obtained by his artificial cultivations in nutritive substances, differing in their nature, of course, from the tubercular substances of the human body. That higher forms of bacteria are really obtained in this manner, has been proved by the researches of Buchner, Zopf and others.

Before closing my remarks on the morphological characters of the bacillus tuberculosis, I cannot forbear to point to the want of correspondence existing in the description of the spores of this bacillus given by different authors. In the passage which I have quoted above from the writings from Koch and Rabes and Cornil on the subject, the reader may, perhaps, already have noticed that this want of correspondence is very great; for while the former regards the granules or minute cells as individual articles of his bacillus tuberculosis in which the spores are contained in the form of *clear, unstained oval bodies*, the latter look upon the *stained cells themselves* as representing the spores, enclosed by the protoplasm of the bacillus. In the first case *the spores do not absorb the staining material*, but remain uncolored and clear in the stained wall of the bacillus—article, in the other the minute cells—which Koch regards as composing the whole body of the bacillus,—are regarded to represent only the spores, and *to be capable of absorbing the staining material*, while the body of the rod—as far as I am able to interpret the meaning of the words of the authors—*remains unstained.*

Watson Cheyne, as may be gathered from the passage concerning the morphology of the bacillus tuberculosis, and which I have above quoted from his writings, speaks, like Koch, of *clear spots* with intermediate stained parts, but, as I have already pointed out before, obscures the meaning of his words by adding that the rod outside of the clear spots is also stained. In examining the illustrations accompanying his paper, however, it will be found that the filaments of the bacillus tuberculosis represented as magnified 2350 diameters in figure 15, do neither show "clear spots" nor "stained rods" outside of these spots.*

Although I have seen in a number of papers these spots of the bacillus tuberculosis mentioned as "clear spots" enclosed in a stained rod, I have never seen them distinctly represented among the illustrative figures accompanying some of these papers but in one instance; I refer to figures illustrating a reprint; also in the *New York Medical Record*, of a paper on "Gram's Method of Staining applied to Pneumonic and Tuberculous Sputum," by M. Afanassiew. Fig. 5 of this paper represents some tuberculous sputum stained by Gram's method

*In referring to the illustrations of Watson Cheyne's paper, I of course take for granted that those accompanying the reprint of his paper in the "New York Medical Abstract," and which I have before me, are correct copies of the original.

and containing numerous bacilli tuberculosis, among which several rods are observed which contain a number of clear, unstained spots or spaces in their interior. As these rods, however, are quite conspicuous among the other ordinary bacilli filaments by being of a greater thickness than these, it appears to me strange that I should never have observed them among the thousands of bacilli tuberculosis contained in the very numerous preparations of stained sputa which I have hitherto examined. But having no reason to suppose that Afanassiew drew these spore-containing rods in his paper without having actually seen them, I am almost inclined to regard them as belonging to another species of bacillus and their presence in this specimen of sputum as accidental.

Besides the discrepancies met with in different descriptions of the spores of the bacillus tuberculosis—some of which I have quoted above—there are other reasons for doubting the real existence of these spores. Thus, we might point to the comparative rarity of the presence of single granules among the hundreds of bacilli tuberculosis filaments contained in a minute portion of tuberculosis sputum or in a thin section of tuberculous lung. For if these spores did really exist in the so-called rod of the bacillus tuberculosis—it matters not whether in the form of Koch's clear, egg-shaped, *unstained* spaces or in the form of Rabes' and Cornil's *stained* granules—they would, in either case, eventually be liberated from the rod and appear among the other bacillus filaments in the form of cocci, and as, according to the views of the above named authors, each bacillus filament, or rod, either contains or represents these spores, they should be present in great numbers in each preparation of tuberculous sputum or lung. This, however, is not the case, for although some cocci forms may be observed in most preparations, they are too few in number to account for a general sporing of all the filaments contained therein.

In closing my remarks on the morphology of the bacillus tuberculosis I may, therefore, repeat that in the tissue of man, at least, I have never met with this organism in the form of a spore-containing rod, but always in the form of a filament composed of minute granules, or cells, and multiplying as it appears by a division of the latter. But as this filament represents morphologically a so called *sphero-bacterium*, I shall follow Zopf in calling it henceforth the "*bacterium tuberculosis.*"

(*To be continued.*)

## Figures and Tables

**Pl. 1 f1:**